# Interventions for Improving Reading Comprehension in Children with ASD: A Systematic Review

**DOI:** 10.3390/bs11010003

**Published:** 2020-12-30

**Authors:** Raúl Tárraga-Mínguez, Irene Gómez-Marí, Pilar Sanz-Cervera

**Affiliations:** Department of Education and School Management, Faculty of Teacher Training, University of Valencia, 46022 Valencia, Spain; irene.gomez@uv.es (I.G.-M.); pilar.sanz-cervera@uv.es (P.S.-C.)

**Keywords:** autism spectrum disorder, intervention, systematic review, reading comprehension

## Abstract

Children with autism spectrum disorder (ASD) often have comorbid learning difficulties in reading comprehension, an essential skill in accessing any area of the curriculum. The aim of this systematic review is to analyze the effectiveness of reading comprehension interventions in students with ASD. We conducted a search for scientific articles published from 2000 to 2019 using the keyword “autis*” in combination with the terms “reading comprehension” and “intervention” or “instruction” in Psyc Info and Scopus databases. After applying inclusion and exclusion criteria, a total of 25 studies were selected. The content analysis of these studies shows that when specific interventions are carried out, students with ASD are able to take advantage of the instruction they receive and compensate for difficulties. Understanding inferences and the main idea of the text are the most common reading comprehension topics, and direct instruction is the most widely-used intervention method in the reviewed studies. Nonetheless, it must be kept in mind that some of the reviews do not specify which sub-processes are addressed in the intervention. Future work should include this aspect, consider the importance of the interventions being implemented by teachers, and take specific aspects of ICT into account that can contribute to improving reading comprehension.

## 1. Introduction

Autism spectrum disorder (ASD) is a neurodevelopmental disorder characterized by the presence of persistent difficulties in communication and social interaction in several contexts, as well as the presence of restrictive and repetitive patterns of behavior, interests, or activities [1]. In addition to these core aspects of ASD, people with this diagnosis usually have other comorbid difficulties that are highly significant in their daily quality of life [2].

One of these common comorbidities consists of learning difficulties in reading comprehension [3,4], probably one of the most relevant academic skills learned in the school context. In fact, reading comprehension in the initial stages of schooling is a good predictor of later academic success and even variables related to behavioral adjustment [5,6,7].

Theoretically, reading comprehension is a complex task that basically requires two phases: the decoding of the graphemes and the extraction of linguistic meanings [8]. Many studies show that people with ASD have difficulties with understanding texts, taking into account their reading decoding ability [9].

In addition, reading comprehension is conditioned by pragmatic characteristics and language comprehension, such as understanding metaphors, jokes, and ironies, making inferences, understanding idioms, or understanding meanings whose interpretation depends on the context. These issues are challenging for students with ASD, even for those with preserved linguistic and cognitive abilities, as in Asperger Syndrome (AS) (level 1 ASD, according to the DSM-5 criteria) [10].

Due to the great heterogeneity in the presentation of the clinical forms of autism, the possible comorbid difficulties, or the age of onset of the first signs and their evolution [11,12], the reading comprehension difficulties can vary in their severity and intensity in students with ASD [13]. Some possible explanations for these difficulties in reading comprehension are the classic theoretical explanations for ASD [14]. For instance, the theory of weak central coherence [15] states that people with ASD have difficulties integrating elements they perceive in isolation into a whole, an essential skill for construction meaning in reading comprehension; the theory of executive dysfunction [16] explains some of the characteristics of ASD based on difficulties in processes such as inhibition, working memory, or planning, key processes in reading comprehension; also, the theory of mind [17] explains some of the difficulties people with ASD have in attributing intentions or mental states in others, a key skill for understanding narrative texts.

Given the reading comprehension difficulties of children with ASD, and considering the relevance of this learning, a large amount of research has been carried out in recent years to evaluate the efficacy of interventions designed to improve reading comprehension in these children.

In this line, a review study of 11 intervention studies published between 1986 and 2006 included seven studies focused on the teaching of vocabulary and four studies aimed at improving text comprehension processes [18]. Another review study [19], which included 11 interventions published between 2000 and 2011 focused on evaluating the effectiveness of computer-assisted interventions. The results of this study showed that computer use can provide effective support for improving reading comprehension in children with ASD through interventions based on solution strategies and making questions related to the comprehension, identification of the structure of the text, and cooperative learning.

A review study that analyzed a total of 12 intervention studies published between 1980 and 2012 [14] found that providing strategies from a cognitive approach, the use of group methodologies, and direct instruction are useful proposals for improving the reading comprehension of students with ASD. Specifically, three of the studies included in this review found that peer tutoring shows benefits in reading comprehension, as well as in social and emotional development. The authors concluded that the implementation of these strategies can be quite beneficial, not only for children with ASD, but also for all students, especially those who present reading and learning difficulties.

Finally, in a review analyzing 15 reading comprehension interventions in students with ASD published between 1989 and 2015 [20], researchers found that only four of the interventions were potentially highly effective, whereas four other interventions obtained an acceptable-high improvement. These interventions agreed on the need to use cooperative learning and graphic organizers, and that it is not advisable to use electronic supports without supervision and it is preferable to design personalized interventions. Additionally, this review also showed that the skills learned in the interventions are transferable to texts that students with ASD face for the first time.

Considering the literature described above, the aim of the present systematic review is to analyze the effectiveness of reading comprehension interventions in students with ASD, considering studies published between 2000 and 2019. Specifically, we aim to answer the following research questions:

1. What results did the educational interventions to improve reading comprehension in children with ASD published between 2010 and 2019 obtain? 2. Which reading sub-processes have these studies focused on? And 3. What are the main characteristics of the interventions regarding methodologies, duration, implementing agents, and context of intervention?

This review expands and updates the previous conclusions of other literature reviews: it covers an updated period of time and includes empirical work carried out to evaluate the effectiveness of any type of reading comprehension intervention (not specific ones). This objective is relevant because it can help to determine which intervention strategies have been effective to improve reading abilities in students with ASD, and so it can help to guide current and future interventions.

## 2. Materials and Methods

### 2.1. Eligibility Criteria

The inclusion criteria used in the review were: (a) empirical studies that evaluate the effectiveness of an intervention to improve reading comprehension; (b) studies that include participants with ASD as the main diagnosis; and (c) studies that include participants between the ages of 5 to 18.

The research was limited to scientific articles published in peer review journals (therefore other types of publications were excluded), from 2000 to 2019.

### 2.2. Information Sources and Search Strategy

The search for and compilation of analyzed articles was carried out through a sequenced research process in the PsycInfo and Scopus databases. We conducted a search for the keyword “autis*” in combination with the terms “reading comprehension” and “intervention” or “instruction”, delimiting any field of the bibliographic record except full text. The first search produced a total of 60 publications in PsycInfo and 100 in Scopus.

### 2.3. Study Selection

**Figure 1:**
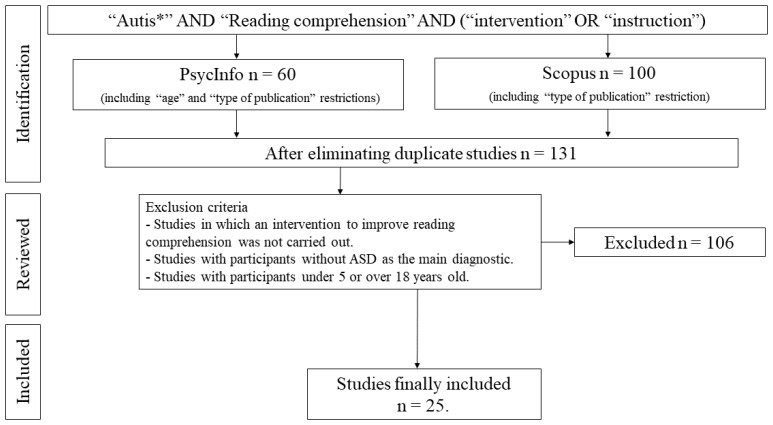
Flow chart of the search process.

After eliminating duplicate studies, two of the authors independently applied the inclusion and exclusion criteria. After that, we included 25 studies in this review. The search and selection process are summarized in Figure 1.

### 2.4. Data Collection Process and Data Items

All 25 studies were independently reviewed by two of the authors of the present review. In each study, they identified: the number, diagnosis, and age of the participants; the reading comprehension subprocesses trained; the type of intervention carried out; the implementer (e.g., teacher, psychologist, or family member) and the context of the intervention (e.g., school, home, or clinic); and a brief summary of the results was obtained. After independent reviews, cases in which there were some divergences in data collection were discussed and resolved by consensus.

## 3. Results

Table 1 includes the information from the 25 selected articles in this review. All of them evaluate the effectiveness of different reading comprehension interventions carried out with children with ASD.

Overall, 196 students from 5 to 17 years old participated in the 25 studies included in this review. Regarding the number of participants: two of the studies were individual case studies [29,37]; most of them (a total of 16 articles) were carried out with two to five participants [21,24,25,26,27,28,30,31,33,34,35,36,39,42,43,45]; in two articles, there were from 10 to 19 participants [32,40]; and, finally, in five studies, there were 20 or more participants [22,23,38,41,44].

Regarding the effect of the interventions, almost all of the studies (except for one [22] that did not obtain significant results), revealed improvements in the reading comprehension skills of students with ASD. Nonetheless, some positive results obtained in different studies were moderate due to the students’ limited language skills [32,33,37,38]. In these cases, reading comprehension skills were conditioned by the low language skills of the participants.

Regarding the specific reading comprehension sub-processes trained in the interventions, some studies worked on various sub-processes at the same time, others worked on only one specific sub-process, and others did not specify the reading comprehension sub-processes included in the intervention. Considering the studies that specify the sub-processes addressed: five of the studies focused on understanding inferences [29,30,34,35,38]; five other studies worked on understanding the main idea [34,38,40,41,43]; four studies focused on identifying the structure and relationship between the elements of the text [27,34,40,41]; three interventions practiced “Wh questions” [21,25,38]; three other studies used comprehension skills related to story elements [26,36,45]; two studies worked on analogies [30,31]; two other studies used paraphrasing [34,38]; one of the interventions focused on understanding metaphors [37]; another study worked on reading fluency and section-based and topography-based comprehension tasks [24]; one study focused on a multicomponent reading comprehension intervention [28]; one study worked on parts of speech, combining sentences with and, identifying contradictions, and identifying relevant/irrelevant information [33]; another study worked on question development and anaphoric cueing [42]; and another study focused on metacognitive and cognitive strategies for improving comprehension [39].

Regarding the type of intervention used: seven studies used direct instruction [27,30,31,32,33,40,41]; six studies used collaborative, guided, or shared reading [21,35,36,39,43,44]; three studies used answering questions [24,25,45]; one study used instruction in the use of digital concept maps of narrative texts read aloud by the researcher [26]; and another study used the Think While After (TWA) strategy [34]. Some studies compared two different interventions: group sessions versus direct instruction [22]; individual sessions versus traditional intervention in school [23]; direct instruction from the teacher versus instruction assisted by a digital tablet [28]; and adding content related to the participants’ persistent interests versus texts without these added contents [29]. Finally, some studies combined several types of activities [37,38,42].

The duration of the interventions varied from 1 to 60 sessions. One study conducted a single session intervention [33]; seven studies conducted 6 to 12 sessions [26,27,34,36,37,42,44]; two studies conducted 13 to 20 [22,45]; four studies conducted 21 to 29 [21,23,29,32]; seven studies conducted 30 sessions or more [24,25,35,39,40,41,43]; and two studies conducted interventions whose duration depended on students’ progress [30,31]. Only two studies did not specify the number of sessions, but the studies lasted four [28] and six [33] weeks. The sessions varied from a mean duration of 10 min to an intensive intervention of 70 min per session.

Regarding the people who implement the intervention, most of the interventions were conducted only by teachers [22,25,27,30,32,33,43,45] or only by researchers [21,23,24,26,28,31,34,35,36,37,38,39,40,41,42,44]. Only one intervention [29] had mixed implementers: researchers and school counsellors.

Finally, the intervention setting was mainly in the school context [21,22,25,26,27,29,30,31,33,35,39,40,41,42,43,44,45]; only three interventions were carried out in clinical settings [34,36,37]; two other interventions took place in a summer camp context [28,32]; and only one intervention was carried out at home [23]. There were interventions that combined two settings: school and University Centre for Autism Research [24]; or school and the home context [38].

## 4. Discussion

In the present study, we have carried out an updated systematic review of empirical studies that analyzed the effectiveness of interventions in different sub-processes of reading comprehension in children with ASD. Specifically, the study raises three research questions.

The first one refers to what results have been obtained by the reviewed research. The main conclusion is that almost all the interventions analyzed produced positive results in the reading comprehension skills of students with ASD. Only one of the 25 studies reviewed did not show significant improvements [22]. This result shows that, when specific interventions are carried out, students with ASD are able to take advantage of the instruction they receive and compensate for difficulties. This result leads to the conclusion that teachers and other practitioners should be encouraged to continue to focus and increase efforts in teaching reading comprehension skills in children with ASD. As shown in this review, these efforts can produce positive short-term results in reading comprehension.

Regarding this, it should be taken into account that advances in reading comprehension were moderated by language skills in some studies. For this reason, language ability became a powerful predictor of reading comprehension [32,33,37,38].

The second research question aims to determine which reading sub-processes have the reviewed studies focused on. Reading comprehension is a complex process involving several sub-processes: vocabulary and syntactic structure knowledge, making inferences, and integration of simple ideas into macro ideas, among others [46]. The results of this review show that understanding inferences and understanding the main idea of the text were the most common reading comprehension sub-processes included in the reviewed interventions. Understanding the main idea of a text is a key point, according to the theory of weak central coherence, which explains that people with ASD have difficulties integrating the elements they perceive in isolation into a whole [10]. Despite these initial difficulties, the present review shows that, if specific strategies are explicitly taught and opportunities for practice are provided, students with ASD are able to extract the main idea from the texts they read.

The third research question refers to several characteristics of the interventions, such as methodologies, duration, implementing agents, and context of intervention.

The results show that a large number of studies used direct instruction, some of them as the only technique [27,30,31,32,33,40,41] and others as a part of the intervention [26]. Direct instruction consists of a teaching approach based on breaking down tasks into sequences of more concrete steps with the aim that students acquire the different skills worked in sequence. It is an approach that emphasizes the structuring of the teaching processes through scripts that guide the teaching process. The results of this review confirm that, according to previous reviews [14,20], this is a positive methodology for teaching school content to children with ASD, considering that these children need individualized attention, and that this systematic methodology is particularly well adapted to the order and structuring needs of students with ASD.

Nevertheless, direct instruction should not be considered as the only option to teach reading comprehension to students with ASD. Collaborative, guided, and shared reading have also been shown as effective methodologies [21,35,36,39,43,44]. These techniques have the value of treating reading as a shared act, highlighting the social value of reading, which can be especially positive for children with ASD. Therefore, given that both direct instruction and shared reading have shown good results, it seems appropriate for practitioners to take both methodological approaches into account when designing educational interventions with their students with ASD, considering the characteristics and particular needs of these students.

Regarding the duration of the interventions, the results show great heterogeneity between the different studies, finding durations from just one session to more than 30. This variety in the length of the interventions is due to the different scope of the objectives of each investigation, as well as the age, skills and previous knowledge of the participants. Reading comprehension training is a process whose results depend to a large extent on reading practice and experience. Therefore, one of the most relevant aspects that we must take into account when designing educational interventions is its ability to be implemented in real contexts. These interventions should have a duration to be feasible over time, considering issues such as the motivation of the students and the availability of time and personal resources to carry them out in natural contexts, either by teachers at school or by families in the home.

If we analyze the ecological validity of the research included in this review, regarding the implementer, the number of interventions carried out by teachers is low (only 7 of the analyzed studies). However, reading comprehension is one of the main forms of learning that take place in school (in fact, it is an instrumental learning, which serves as a base tool for later learning). So, it is necessary to design educational interventions that, once shown to be effective in the field of research, could be applied in schools with the usual resources in this context. To transfer the results of the research to the school environment, it is important to consider: the skills and teachers’ resources, the ratio of students per classroom, and the possibility of implementing interventions following the schools’ schedules.

Some studies have taken these aspects into account, since the interventions have been carried out in school contexts by the teachers (not by researchers) throughout all the research process or at least in a generalization phase [22,25,27,30,43,45]. These investigations provide added value to the field, since they furnish greater confidence about the ecological validity of the results obtained.

Nonetheless, there is a large amount of research that has been conducted in different settings other than the school context. In these cases, these educational interventions have demonstrated its effectiveness in improving the reading comprehension of children with ASD under controlled conditions. However, we cannot reliably affirm that teachers in the typical conditions of their classrooms can apply these same intervention procedures carried out in control conditions.

In fact, we found two studies in this review that have used the same intervention procedures (based on ABRA, a free computer-assisted literacy program) in two different contexts. In a first study [23], ABRA intervention was conducted by a researcher on a 1:1 basis in participants’ homes, obtaining good results also in reading fluency as in reading comprehension. In a later study [22], ABRA was conducted by teachers at school in a more naturalistic context, obtaining also good results in reading fluency, but not in reading comprehension. The possibility of comparing these two studies, which apply the same intervention in two different settings, obtaining different results, gives us the opportunity to reflect on the importance of designing interventions that can be put into practice in the usual school conditions.

In addition to the necessary school and teacher involvement, reading is an activity that can be carried out at home as a dyadic activity with families. Reading at home with parents can have benefits not only in improving reading skills, but also in improving joint attention and certain aspects related to communication and social interaction [47,48]. For this reason, in addition to designing interventions to carry out in the school context, future research should design interventions that can be implemented in the home context.

Finally, regarding students’ motivation towards reading, this research has shown that some tools and strategies used to carry out interventions are interesting and could be attractive to children with ASD. That is the case of ICT support [21], the ABRA computer assisted intervention [22,23], and the inclusion of content according to the students’ interests [19], among other initiatives. Additionally, it is important to consider the use of maps and graphic organizers as tools to enhance the comprehension of text information. In fact, in the articles reviewed that used these aids [26,27,35,45], and according to a previous review study [20], the results were highly effective and the improvements in reading comprehension were more noteworthy, compared to studies that did not use visual aids.

### 4.1. Limitations of the Study

Conclusions about the effectiveness of the interventions analyzed in the present review should be viewed with caution because they could be influenced by publication bias. The 25 studies included in this review have been published. Nevertheless, this review does not include any unpublished studies. Because interventions that do not obtain good results usually remain unpublished, not including any of these studies could skew the conclusions about the effectiveness of these interventions.

In addition, reading comprehension is a complex task that includes a large variety of cognitive and meta-cognitive sub-processes. Some of the studies included in this review did not specify which sub-processes were addressed in their interventions, and most of the studies in which the sub-processes were specified included many different types of activities. Therefore, it is difficult to determine what specific types of interventions are more effective. Future studies should analyze the effectiveness of the interventions while considering the sub-processes that were worked on in each case in greater depth.

### 4.2. Future Research

Future lines of inquiry should carry out research to identify which specific aspects cause the greatest difficulties for children with ASD, and continue to investigate specific aspects of ICT that can contribute to improving the reading comprehension of children with ASD. These interventions should be implemented by teachers in their ordinary school environment in order to be considered valid.

## 5. Conclusions

Through our review, we were able to provide an overview of different interventions carried out with students with ASD, confirming that they are effective strategies to improve the reading comprehension of children with ASD. Among the most used intervention strategies, two methodologies with very different characteristics stand out. One of them is direct instruction, a methodology characterized by a high structure, and the existence of exhaustive scripts on the actions to be carried out by teachers and students. The other one that we found was the collaborative, guided and shared reading methodologies, characterized by highlighting the social and communicative aspects of reading.

Both methodological approaches can be considered as being effective, although in different ways. While direct instruction emphasizes mastery of purely cognitive aspects, shared reading brings into play issues of a much more social nature. Since both facets of reading are important, teachers and professionals who are responsible for teaching reading must be aware of the strengths and weaknesses of each method to make well-informed decisions about which strategies employ taking into account the set objectives and the students’ characteristics.

We think this review may be of interest to both researchers and teachers who want their pupils with ASD to improve their reading comprehension skills. Finally, we hope that our work will lead to further research and better and more useful practices.

## Figures and Tables

**Table 1:** Analysis of the studies included in the review.

Author (s)	Participants	Sub-Process of Reading Comprehension	Intervention	Implementer and Context	Results
[21]	N = 3 ASD 8, 8 and 10 years old	“Wh questions” comprehension	Shared reading with ICT support and answer questions. 21 sessions	School Researcher	Reading shared stories with ICT support facilitated the understanding of “Wh questions”
[22]	N = 23 ASD 5.83–8.42 years old	Reading accuracy and reading comprehension	ABRACADABRA (ABRA) video game. ABRA comparison in group sessions vs. direct instruction. 20 sessions	School Teachers	Group use of ABRA in the school context improves reading accuracy, but not reading comprehension
[23]	N = 20 ASD, AS, GDD 5–11 years old	Reading accuracy and reading comprehension	ABRA video game. ABRA comparison in individual sessions vs. traditional intervention in school. 26 sessions	Home Researchers	Individual use of ABRA produces significant improvements in both accuracy and reading comprehension
[24]	N = 3 ASD and GDD 11–12 years old	Reading fluency and section-based and topography-based comprehension tasks	Reading followed by 10 non-inferential questions per passage. 31 sessions of 30–70 min each	School and University Centre for Autism Investigator	No evidence for improvements in fluency; but there is evidence for improvements in reading comprehension
[25]	N = 3 ASD 8–10 years old	“Wh questions” comprehension	Practice answering “Wh questions”. 30 sessions of 10 min each	School Teachers	Improvements in the baselines of the three students’ accuracy in the responses to “Wh questions”
[26]	N = 3 ASD 8–10 years old	Comprehension skills related to story elements, including: graphic organizers, story structure, question answering, and multiple strategy instruction	Instruction in the use of (digital) concept maps of narrative texts read aloud by the researcher. 6–12 sessions of 20–30 min	School Researcher	The use of concept maps was effective for understanding narrative texts
[27]	N = 3 ASD 15–16 years old	Reading comprehension. Identification of text structure	Direct instruction. Use of graphic organizers, analysis of text structure, and text prediction. 8 sessions	School Teachers	Highly effective instructions during the intervention and the follow-up
[28]	N = 3 AS 9–11 years old	Multicomponent reading comprehension intervention, including: teaching text preview strategy, identifying the main idea of each paragraph using a graphic organizer, and the use of a token economy system	Comparison of direct instruction from the teacher vs. instruction assisted by digital tablet. 4 weeks	Summer camp Researchers	Teacher support is more effective and shows better results than using the i-Pad
[29]	N = 1 ASD 8 years old	Literal information and inferences	Comparison reading of texts in which they add content related to the persistent interests of the participant vs. texts without these added contents. 22 sessions of 30 min each	School Researcher and school counselling	Introducing content related to the interests of students with ASD improves their reading comprehension results
[30]	N = 4 ASD 10–14 years old	Inference of statements, use of facts and analogies	Direct instruction in reading comprehension. 1–4 sessions of 20 min per week	School Teachers	Improvement in inferences, use of analogies in the four students. Maintenance after one month without specific intervention
[31]	N = 2 ASD 11–14 years old	Analogies, induction, and deductions	Direct instruction in reading comprehension. 1–4 sessions of 20 min per week	School Researcher	Positive results for the four students on analogies and deductions, and in three students for inductions.
[32]	N = 18 (11 with ASD; 7 with intellectual disability) 7–13 years old	Reading comprehension	Direct instruction in reading comprehension and language programs. 25 sessions of 30 min each	Summer school Teachers	Both interventions show significant improvements in students’ skills throughout the process
[33]	N = 2 ASD 10–16 years old	Parts of speech, combining sentences with and, identifying contradictions, and identifying relevant/irrelevant information	Program for correcting the answers to comprehension questions. Direct instruction. 6 weeks	School Researcher	Improvement in the reading comprehension of each participant
[34]	N = 4 ASD 10–11 years old	Paraphrasing of texts and understanding structure, explicit information, making inferences, main idea, vocabulary, and syntax	Think While After (TWA) strategy: think before reading, while reading and after reading. 6 sessions of 45 min each	Researchers	Effective or very effective intervention in improving understanding after the intervention and in follow-up evaluation
[35]	N = 2 ASD 5 years old	Comprehension of literal and inferential questions in science texts	Shared reading and use of conceptual maps. 32–60 sessions of 20 min each	School Researcher	The intervention was effective for understanding literal information and making inferences.
[36]	N = 3 ASD 6–8 years old	Reading questions on narrative story comprehension	Shared reading. Comprehension strategies before, during, and after reading (order sequences, summarize, paraphrase, and answer questions). 6–7 sessions of 28 min each	Autism Clinic Researcher	All the participants demonstrated remarkable improvements in reading comprehension. The improvements endured during monitoring phase
[37]	N = 1 ASD 10 years old	Metaphor comprehension	Three types of activities: identify comparisons, rename concepts, and continue a story that ends with a metaphorical meaning. 6 sessions of 60 min each	Research University Clinical Centre	Improvement in understanding sensory metaphors. Limited progress in understanding psychological metaphors.
[38]	N = 20 (10, ASD; 6, AS; 4, GDD) 14–17 years old	Comprehension of narrative texts: main idea, reformulate ideas, inferences, answer questions	Three types of comprehension tasks: cloze tasks; understanding anaphoric inferences; and prior knowledge questions about reading. 1 session of 60 min	School and home Researcher	The use of aids to detect anaphoric inferences was more effective in improving understanding than the other two interventions.
[39]	N = 3 ASD 15–17 years old	Metacognitive and cognitive strategies for improving comprehension	Collaborative reading (in pairs). 2–3 sessions of 30 min each for 16 weeks	School Researcher	Improvements in reading comprehension in the three cases
[40]	N = 13 ASD high functioning 9 years old	Identification of the main idea, identification of anaphoric relationships	Direct instruction in small group. 30 sessions of 30 min each	School Researcher	Progress in vocabulary, identification of main ideas, and reading comprehension
[41]	N = 21 ASD, GDD and AS. 6–12 years old	Identification of the main idea, identification of anaphoric relationships, and vocabulary	Direct group instruction. 3 sessions during a period of 16 weeks	School Researcher	Students with high functioning autism clearly benefited from the intervention
[42]	Study 1 N = 2 ASD 12 and 13 years old Study 2 N = 2 ASD and AS 10 years old	Question development and anaphoric cueing	Study 1 Training in creation of literal questions + applied behavioral analysis (ABA). 8 sessions of 30 min each. Study 2 Training in anaphora solution + ABA. 9 sessions of 30 min each	School Researcher	Interventions that combine ABA with a reading comprehension intervention are better than interventions focused only on comprehension
[43]	N = 5 ASD 12–14 years old	Vocabulary and main idea intervention	Guided reading of journalistic texts. In certain sessions, the student was allowed to choose the text to work on. 37 or 40 sessions of 20–30 min each	School Teachers	Improvements in the level of reading comprehension and vocabulary.There were no differences in understanding between the texts chosen by the students and those not chosen by them
[44]	N = 29 ASD and AS 11–15 years old	Non-verbal reasoning ability, expressive vocabulary, accuracy, speed, and reading comprehension	Collaborative reading to work on: prediction, explain the meaning of terms, ask questions about the plot and the characters, and summarize some excerpts from the book. 12 sessions of 45 min each	School Researcher	Significant improvement in reading comprehension. Transfer of learned skills to other areas of the curriculum
[45]	N = 3 ASD 13–17 years old	Narrative texts comprehension	Reading and listening to the text, followed by work on a character action map and 10 comprehension questions. 20 sessions (including pre- and post-intervention)	School Teachers	Improvement in the reading comprehension of the three characters

## Data Availability

No new data were created or analyzed in this study. Data sharing is not applicable to this article.
